# Obituary

**Published:** 1892-11

**Authors:** 


### Obituary.

Dr. Thomas Fanning Wood, senior editor of the *North Carolina Medical Journal*, died in Wilmington, August 22, 1892, in the fifty-second year of his age. Dr. Wood was one of the most accomplished physicians in the South. He had been active in securing the enactment of the law providing for the State Examining and Licensing Board, and was one of its most prominent and active members for some years. He was also a member of the State Board of Health, and was Vice-President of the American Public Health Association in 1891.

Dr. Wood was a student of botany, and found recreation in pursuing this interesting field. He left a large library, with many works in it pertaining to his favorite subject, and a herbarium which is one of the most complete of its kind. The various societies with which he was connected have passed memorials that bespeak in touching terms of their affection for their departed friend and colaborer. It is not often so marked a man dies in the midst of his activity, and we beg to join our North Carolina confrères in their tributes of respect to the memory of one who was such a distinguished ornament to our profession.

---

Dr. Walter Coles, of St. Louis, died August 9, 1892, after a long and trying illness that depressed his spirits to that degree which compelled him to seek his own destruction. Dr. Coles was a well-equipped man in several respects, but had particularly distinguished himself in the field of obstetrics and gynecology, in which department of medicine he had taught successfully and practised faithfully. Among the many physicians who have fallen during the year, Dr. Coles's name will stand prominently. The St. Louis medical magazines have published fitting tributes to his memory, and the *St. Louis Medical and Surgical Journal* has issued a most excellent half-tone portrait of the deceased. His funeral was largely attended by a sorrowing profession.

